# Association of the *FCRL3 *gene with rheumatoid arthritis: a further example of population specificity?

**DOI:** 10.1186/ar2006

**Published:** 2006-07-19

**Authors:** Stephen Eyre, John Bowes, Catherine Potter, Jane Worthington, Anne Barton

**Affiliations:** 1 ARC-EU, University of Manchester, UK

## Abstract

Association of a functional promoter polymorphism mapping to the Fc receptor-like 3 (*FCRL3*) gene has recently been reported and replicated with rheumatoid arthritis (RA) in Japanese populations. The aim of this study was to investigate association of the FCRL3 gene with RA in UK subjects. DNA was available from 1065 patients with RA and 2073 population controls from the UK. Four single nucleotide polymorphism (SNP) markers (FCRL3-169*C/T (fclr3_3, rs7528684), fclr3_4 (rs11264799), fclr3_5 (rs945635), fclr3_6 (rs3761959)) all previously associated with RA in a Japanese population were genotyped in 761 RA samples and 484 controls. In the remaining samples, only the putative disease causal polymorphism, FCRL3-169*C/T, was tested. Genotyping was performed using either the Sequenom MassArray iPlex platform or a 5' Allelic discrimination assay (Taqman, ABI). Extensive linkage disequilibrium was present across the promoter SNPs genotyped (r^2^ values = 0.60-0.98). Allele frequencies did not differ between RA cases and controls either for the putative disease causal polymorphism (odds ratio FCRL3-169*C allele = 0.97 (0.87-1.07), p = 0.51) or for the other SNPs tested. Similarly, no association was detected with RA using haplotype analysis or when stratification by shared epitope carriage or by presence of rheumatoid factor was undertaken. This study was powered to detect an effect size of 1.24 or greater for the FCRL3-169*C/T functional promoter polymorphism but no evidence for association was detected, suggesting that this gene will not have a substantial effect in determining susceptibility to RA in populations of Northern European descent.

## Introduction

Rheumatoid arthritis (RA) is a complex disease, the hallmark of which is synovial joint inflammation. The heritability of RA has been estimated to be in the order of 60%, suggesting a substantial contribution from genetic factors [1]. The major susceptibility gene is the *HLA DRB1 *gene. Carriage of certain alleles, collectively termed shared epitope alleles, confers a twofold to threefold increased risk of RA.

Evidence for association with functional variants in two other genes has been confirmed in multiple populations. Firstly, the association of the PTPN22*R620W gene with RA in Caucasians of Northern European descent has been widely replicated (summarised in [2]). Interestingly, this disease causal polymorphism is not present in the Japanese population, and haplotype analysis of other polymorphisms mapping to the gene has revealed no evidence for association [3]. Secondly, association with a functional haplotype of the *PADI4 *gene has been consistently demonstrated, mainly in Far Eastern populations [4-6].

An association with a further putative susceptibility gene has recently been reported. Association with a functional promoter variant of the Fc receptor-like 3 (*FCRL3*) gene has been detected and replicated in Japanese patients with RA [7]. The gene maps to 1q21, and linkage analysis studies in Japanese RA families have previously highlighted this region as potentially harbouring a susceptibility gene. Fine mapping under the region of linkage identified association with RA of a promoter polymorphism of the *FCRL3 *gene, and the association was confirmed in an independent replication case–control cohort, again of Japanese descent.

The *FCRL3 *gene has structural homology with the classical FcγRs, and the protein product was shown to be expressed in B cells, in secondary lymphoid organs and in aggregates of lymphocytes in synovial tissues from RA patients. It has been demonstrated that the associated promoter polymorphism (FCRL3-169*C/T) was shown to affect expression through NF-κB binding. Furthermore, the number of FCRL3-169*C susceptibility alleles correlated with autoantibody (rheumatoid factor and cyclic citrullinated peptide antibodies) levels and was higher in RA patients with two copies of shared epitope alleles.

The association of a functional promoter polymorphism in a strong candidate gene expressed in appropriate tissues has therefore been detected and replicated with RA in a Japanese population. It has already been noted, however, that striking differences in associations have been found between Caucasians of Northern European descent and Far Eastern populations for the non-HLA RA susceptibility genes identified to date. Hence, the aim of the current study was to assess whether the same *FCRL3 *genepolymorphisms were associated with RA in a UK population.

## Patients and methods

### Study design

A case–control (association) study was performed comparing genotype frequencies of single nucleotide polymorphisms (SNPs) mapping to the *FCRL3 *gene, previously associated with RA in a Japanese population, with UK RA patients and controls. Genotyping was performed in two stages: in the first phase, association was tested with four SNPs in a subset of the total cohort; while in the second stage, only the SNP identified as the probable disease causal polymorphism in the Japanese study (FCRL3-169*C/T (fclr3_3, rs7528684)) was genotyped in the remaining samples [7]. Comparisons with controls were made in the case group as a whole and in subgroups stratified by carriage of the *HLA DRB1 *shared epitope and PTPN22*620W alleles. Haplotypes were estimated using the EM algorithm implemented in HelixTree software (Golden Helix Inc., Bozeman, MT, USA) and frequencies were compared between cases and controls.

### Subjects

DNA was available from 1,065 cases with RA. All cases satisfied American College of Rheumatology 1987 classification criteria for RA modified for genetic studies [8] and were recruited as described previously [9]. Of these participants, 65.3% (690/1057) were female, 77.2% (782/1013) were rheumatoid factor-positive, 79.7% (642/806) were erosive, and 20.4% (149/729), 49.1% (358/729) and 30.5% (222/729) patients carried zero, one or two copies of shared epitope alleles, respectively.

Control subjects with no history of inflammatory arthritis were recruited from blood donors and general practitioner registers (*n *= 484) or from subjects recruited as part of the 1958 Birth Cohort, a cohort comprising DNA samples from a randomly selected subset of children born in England, Scotland, and Wales in 1 week in March 1958 (approximately 17,000 live births) who have been followed prospectively [10]. The Oversight Committee for the Biomedical Assessment of the British 1958 Birth Cohort Study provided access to DNA collected from 2,064 individuals from the 1958 birth cohort randomly distributed across the United Kingdom. Genotype data generated from the 64 non-White individuals included in the cohort was not included in the analysis. Of the available samples, genotyping was attempted in 1,600 and was successful in 1,589 in the current study. Of all the controls genotyped, 1,144/2,073 were female (55.2%). *HLA DRB1 *data were available only on a subset but, of these, 50.4% (605/1200), 39.4% (473/1200) and 10.2% (122/1200) carried zero, one or two copies of shared epitope alleles, respectively.

Ethical approval for the study was obtained and all participants provided informed consent.

### Polymorphisms selected

Four SNPs – FCRL3-169*C/T (fclr3_3, rs7528684), FCRL3-169*C/T (fclr3_4, rs11264799), FCRL3-169*C/T (fclr3_5, rs945635) and FCRL3-169*C/T (fclr3_6, rs3761959) – were initially selected for investigation because they had all been associated with RA in the Japanese population on single-point analysis, because the SNPs formed a haplotype associated with RA and because the most probable disease causal SNP (FCRL3-169*C/T (fclr3_3, rs7528684)) was included [7].

### Genotyping

Genotyping was performed mainly using the Sequenom MassArray platform according to the manufacturer's instructions [11]. For one SNP, FCRL3-169*C/T (fclr3_3, rs7528684), a subset of the samples were genotyped using a 5'-allelic discrimination assay as described previously [9]. Duplicate samples and negative controls were included across the plates to ensure accuracy of genotyping. In addition, for all the assays, genotyping was confirmed in a subset of samples using the Pyrosequencing genotyping method according to manufacturer's instructions [12].

### Haplotype analysis

Haplotypes were estimated using the EM algorithm implemented in HelixTree software (Golden Helix Inc.). Haplotypes were also estimated using the PHASE program, which uses the Markov Chain Monte Carlo method to estimate haplotypes [13]. Missing genotypes were imputed and estimated haplotype frequencies compared between RA cases and controls.

### Power

The study had 80% power to detect an odds ratio of 1.24 at the 5% significance level, assuming a recessive model [7]. It was powered to detect a smaller effect size than that originally reported in the Japanese study (odds ratios of 2.15 and 1.3 for the test cohort and replication cohort, respectively) as it is accepted that effect sizes are often overestimated in first studies.

## Results

Re-genotyping all SNPs in a subset of samples using the pyrosequencing platform revealed 100% concordance between genotype calls made from different platforms. A subset of samples genotyped using the Sequenom and Taqman (ABI, Warrington, UK) platforms for the FCRL3-169*C/T (fclr3_3, rs7528684) SNP were also 100% concordant. Duplicate samples across plates similarly showed no discrepant calls.

No deviation from Hardy–Weinberg expectations was observed for any of the SNPs in either cases or controls. Interim analysis after genotyping a subset of the total cohort revealed that extensive linkage disequilibrium was present across the four SNPs tested. Indeed, the *D' *value between all the SNPs was 0.98 while the correlation between SNPs FCRL3-169*C/T (fclr3_3, rs7528684), FCRL3-169*C/T fclr3_5 (rs945635) and FCRL3-169*C/T fclr3_6 (rs3761959) was also 0.98 in this sample. The correlation between these SNPs and FCRL3-169*C/T fclr3_4 (rs11264799) was 0.6 or greater, reflecting the difference in allele frequency of this SNP compared with the others. Hence, genotyping of all four SNPs was unnecessary and, in the remaining samples, only the putative disease causal polymorphism was genotyped.

None of the SNPs tested was associated with RA using single-point analysis methods (Table 1). By contrast to the findings in the Japanese study, we found no correlation of FCRL3-169*C susceptibility allele frequency with shared epitope allele carriage or with the presence of rheumatoid factor (Table 1). Stratification based on carriage or absence of shared epitope alleles (data not shown), on carriage of the PTPN22*620W gene susceptibility variant (odds ratio FCRL3-169*C allele = 1.12 (95% confidence interval 0.87–1.44), *P *= 0.37), on gender and on age at onset of RA similarly revealed no evidence for association of the SNPs in these subgroups (data not shown).

### Haplotype analysis

Of the 16 possible estimated haplotypes, only three existed at a population frequency greater than 1%. Estimated frequencies were similar whether estimated using the EM algorithm in HelixTree software (Golden Helix Inc.) or the Markov Chain Monte Carlo algorithm in the PHASE program [13]. No difference in the distribution of these haplotypes was noted between cases and controls (Table 2).

## Discussion

Association of a promoter polymorphism mapping to the *FCRL3 *gene with RA has recently been reported in a Japanese population [7]. The gene is likely to play a role in immune regulation, and functional data showed that the associated polymorphism controls the levels of gene expression. The association was replicated in the same study in another cohort of RA patients, with systemic lupus erythematosus and autoimmune thyroid disease. Furthermore, association has since been replicated by a separate group of investigators in an independent cohort of RA patients and controls from Japan [14]. Hence, strong evidence exists to support the hypothesis that variation within the gene may be disease causal. In this well-powered study, however, we have failed to demonstrate a similar association with RA in a UK population.

Our result may represent a false-negative finding, but this is unlikely for several reasons. Firstly, we had 100% power to detect a similar effect size to that reported in the original study (effect size in test group, 2.15) and 90% power to detect the effect size of 1.3 reported in their replication cohort [7]. Secondly, false-negative findings can also occur as a result of population stratification, but our control allele frequencies are similar to those reported previously in populations of Northern European descent and no difference in allele frequencies was observed between control or case samples from different regions of the United Kingdom (data not shown). Finally, previous studies from US and Spanish populations have also failed to demonstrate association with RA at this locus [15,16].

It seems probable, then, that the association of this polymorphism with RA may be population specific. This is analogous to the association of the PTPN22*R620W polymorphism with RA in European, Northern and Southern American populations but not in Far Eastern populations (summarised in [2]). In this case, the population specificity arises from the fact that the polymorphism is common in European and Northern American populations (approximately 10% minor allele frequency) but very rare in Japan. For the *FCRL3 *gene, however, the putative disease causal allele is common in both the UK and Japanese cohorts (45% and 35% minor allele frequency, respectively) so this cannot explain the population differences found. Similarly, the same haplotypes exist, albeit at slightly different frequencies.

Association of the *PADI4 *gene with RA also shows variation between populations and, like the FCRL3-169*C/T gene polymorphism, the susceptibility haplotype is common in all populations. A functional haplotype of the *PADI4 *gene has been consistently associated with RA in Far Eastern populations [4-6]. While most studies in patients of Northern European descent have failed to demonstrate any association [9,17,18], one study in a US population did recently report that this gene may have a very small effect [19]. This finding has yet to be confirmed but, if it is, it may be argued that the effect size for the *FCRL3 *gene may also simply be much smaller in populations of Northern European descent, possibly reflecting differences in exposure to environmental susceptibility factors between populations. In support of this, a study in the UK population has replicated association of the FCRL3-169*C/T gene polymorphism with Graves' disease but with a smaller effect size [20]. Our study was sufficiently powered, however, to detect an effect size of 1.24. Indeed, by combining our data with those reported from a North American population, an effect size of 1.18 can be excluded with 80% power [15].

The differences in the association of disease in different populations highlights the importance of accounting for ethnic origin when performing association studies, particularly in populations where considerable ethnic mixing may have occurred, such as that found in the United States.

## Conclusion

The presented findings do not support a major role for the *FCRL3 *gene in determining susceptibility to RA in populations of Northern European descent.

## Abbreviations

*FCRL3 *= Fc receptor-like 3; NF = nuclear factor; RA = rheumatoid arthritis; SNP = single nucleotide polymorphism.

## Competing interests

The authors declare that they have no competing interests.

## Authors' contributions

SE, JW and AB contributed to the study design. SE, CP and JB generated the data. SE and AB undertook the analysis. SE, JW and AB participated in the preparation of the manuscript.

## Figures and Tables

**Table 1:** Genotype frequencies of Fc receptor-like 3(*FCRL3*) single nucleotide polymorphisms in rheumatoid arthritis cases, in controls and in subgroups

Single nucleotide polymorphism	Controls	Cases	Two-copy shared epitope alleles^b^		Rheumatoid factor-positive ^a, c^
				
			Controls	Cases	
FCRL3-169*C/T (fclr3_3, rs7528684)					
T/T	595 (28.7)	324 (30.4)	45 (36.9)	71 (32.0)	324 (30.4)
C/T	1055 (50.9)	524 (49.2)	52 (42.6)	103 (46.4)	524 (49.2)
C/C	423 (20.4)	217 (20.4)	25 (20.5)	48 (21.6)	217 (20.4)
*P *value	0.57		0.69		0.57
FCRL3-169*C/T (fclr3_4, rs11264799)					
1/1	258 (53.3)	404 (53.1)	13 (50.0)	132 (52.6)	404 (53.1)
1/2	190 (39.3)	296 (38.9)	10 (38.5)	95 (37.8)	296 (38.9)
2/2	36 (7.4)	61 (8.0)	3 (11.5)	24 (9.6)	61 (8.0)
*P *value	0.93		0.94		0.93
FCRL3-169*C/T (fclr3_5, rs945635)					
1/1	123 (27.9)	209 (28.4)	9 (36.0)	64 (27.3)	209 (28.4)
1/2	200 (45.3)	362 (49.3)	12 (48.0)	116 (49.6)	362 (49.3)
2/2	118 (26.8)	164 (22.3)	4 (16.0)	54 (23.1)	164 (22.3)
*P *value	0.46		0.57		0.46
FCRL3-169*C/T (fclr3_6, rs3761959)					
1/1	124 (26.3)	209 (27.8)	8 (30.8)	65 (27.1)	209 (27.8)
1/2	220 (46.7)	370 (49.3)	12 (46.1)	120 (50.0)	370 (49.3)
2/2	127 (27.0)	172 (22.9)	6 (23.1)	55 (22.9)	172 (22.9)
*P *value	0.28		0.91		0.28

Data presented as *n *(%). ^a^Comparison group = all controls. ^b^Subgroup stratified by two copies of shared epitope alleles. ^c^Subgroup stratified by the presence of rheumatoid factor (measured cumulatively).

**Table 2:** Estimated haplotype frequency distributions in cases and controls

Haplotype	Controls (%)	Cases (%)	Overall comparison (*P *value)
T, G, G, G	53.1	52.7	0.97
C, A, C, A^a^	25.2	27.2	
C, G, C, A	20.6	18.5	

^a^Haplotype associated with rheumatoid arthritis in a Japanese population.
